# Immunohistochemical Detection of Cancer Stem Cell Related Markers CD44 and CD133 in Metastatic Colorectal Cancer Patients

**DOI:** 10.1155/2014/432139

**Published:** 2014-04-22

**Authors:** Pavel Pitule, Miroslava Cedikova, Ondrej Daum, Jan Vojtisek, Ondrej Vycital, Petr Hosek, Vladislav Treska, Ondrej Hes, Milena Kralickova, Vaclav Liska

**Affiliations:** ^1^Biomedical Center, Faculty of Medicine in Pilsen, Charles University in Prague, Husova 3, 306 05 Pilsen, Czech Republic; ^2^Department of Histology and Embryology, Faculty of Medicine in Pilsen, Charles University in Prague, Karlovarská 48, 301 00 Pilsen, Czech Republic; ^3^Sikl's Department of Pathology, Medical School and Teaching Hospital Pilsen, Charles University in Prague, Dr. E. Benese 13, 305 99 Pilsen, Czech Republic; ^4^Department of Surgery, Medical School and Teaching Hospital Pilsen, Charles University in Prague, Alej Svobody 80, 304 60 Pilsen, Czech Republic

## Abstract

*Aim*. The goal of this study was to semiquantitatively detect presence of cancer stem cells markers CD44 and CD133 in immunohistochemically stained paired samples of colorectal cancer (CRC) and colorectal liver metastases (CLM). Level of staining intensity was compared to clinical and pathological characteristics of tumors with the aim to identify impact of CD44 or CD133 expression on tumor behavior. *Patients and Methods*. Formalin fixed paraffin embedded samples from 94 patients with colorectal tumor and liver metastases were collected at Sikl's Department of Pathology. Samples were stained by antibodies against CD44 and CD133. Presence and intensity of staining was assessed semiquantitatively by three trained researchers. *Results*. Patients with higher level of CD133 staining in CRC had longer disease free interval (Cox-Mantel *P* = 0.0244), whereas we found no relation between CD44 expression and overall survival or disease free interval. CD133 expression in CRC and CLM differed based on CRC grading; in case of CD44 we found differences in staining intensity in individual stages of tumor lymph node invasion. *Conclusion*. Effect of cancer stem cell markers on prognosis of colorectal cancer can vary depending on pathological classification of tumor, and we have shown that CD133, generally considered to be a negative marker, can bear also clinically positive prognostic information in group of patients with colorectal liver metastases.

## 1. Introduction

For a long time, tumors were considered to be unhierarchical cell mass, sometimes with several clonal populations, where all cells had almost the same potential for development, growth, or secondary tumor formation. The last two decades brought new findings and tumors have gradually become regarded as hierarchical tissues, similarly to normal tissues, with different cell populations, each having a distinct function and characteristic within the tumor. One minor population is of particular importance—small percentage of tumor cells called cancer stem cells (CSCs).

Hierarchical organization of tumors was for the first time identified in acute myeloid leukemia in 1997 [1], where cells with CD34^++^CD38^−^ phenotype were described as primitive leukemic stem cells with the potential to differentiate into the leukemic blasts. Similar subpopulation with low level differentiation was later described also in many different solid tumors, including breast [2], prostate [3], colon [4, 5], or pancreatic cancer [6]. Today, there are two main models describing involvement of CSCs in tumor development: deterministic, according to which all tumor cells arise from CSCs, which are dividing asymmetrically and stochastic, which supposes the tumor cells to be randomly acquiring mutations and undergoing clonal evolution that can result in the formation of a clone with stem-cell properties. The latter model assumes that the cells are dividing symmetrically [7].

Cancer stem cells share many similarities with physiologically normal adult stem cells. Both of these cell types are undifferentiated with the capacity to differentiate into hierarchical sequence of other tumor or normal cells; they are capable of self-renewal and asymmetric division and they have relatively long cycling times and long-term survival [8]. In addition to this, CSCs were described to be highly resistant to chemotherapy and radiotherapy which makes them very difficult to target and eliminates them by common therapy regimens. This characteristic makes them a possible source of later recurrence of the disease or therapy-driven selection of resistant clones [9]. Several treatment regimens specifically targeting CSCs are now emerging to overcome this problem. One of these approaches is forced differentiation of CSCs combined with targeted therapy [10].

During the initial attempts to identify CSCs, tumor cells were usually separated according to the expression of a particular marker into two groups (positive and negative). The capacity of the cells to form new tumors was then assessed and compared between the two groups. Using this approach in colorectal cancer, it was described that cells expressing CD133 are more tumorigenic than CD133 negative ones [4, 5]. Later on, additional markers were described, for example, CD44 and CD166 [11], CD29, CD24, and Lgr5 [12], and ALDH1 [13]. Many of these markers are also expressed in normal colonic stem cells (e.g., Lgr5, ALDH1, or CD29), which complicates the distinction between CSCs and normal stem cells. There are also other discrepancies regarding these markers. It was described, for example, that cells with stem cell capacity also exist within the CD133 negative cell population and that CD133 negative cells can form tumors with the same frequency as CD133 positive cells [14]. Further studies are necessary to identify the characteristics of cancer stem cells more precisely, because their unbiased identification and understanding of their biology can open new options for cancer treatment.

In the presented study, we selected two putative cancer stem cell markers, CD44 and CD133, to compare their expression in matched primary colorectal tumor and colorectal liver metastases within clinically well-specified set of patients. We evaluated the relationship between markers expression in primary and secondary tumor and tested the impact of CD44 and CD133 positivity on clinical behavior of tumor, mainly on overall survival and disease free interval.

## 2. Methods

Assessment of positivity or negativity for CD44 and CD133 was performed semiquantitatively from immunohistochemically stained sections of matched primary and secondary tumor samples from patients with colorectal carcinoma and either synchronous or metachronous liver metastasis. Immunohistochemical staining was selected as a method of choice, because it is a commonly used technique in many pathology departments and new markers can be easily implemented to current protocols.

### 2.1. Selection of Samples

Samples used for this study were collected from the depository of formalin fixed paraffin embedded samples of Sikl's Department of Pathology, Medical School and Teaching Hospital in Pilsen. We have selected patients who underwent surgery for primary colorectal cancer between years 1996 and 2010 and who were afterwards subjected to the surgery for colorectal cancer liver metastases at Department of Surgery, Medical School and Teaching Hospital in Pilsen.

The data required to determine overall survival and disease free interval, as well as grading and staging (TNM classification) scores, were available from the clinical information system of Teaching Hospital in Pilsen. The patients' data were anonymized by authorized medical personnel before being processed. Description of the patient sample is summarized in Table 1.

### 2.2. Immunohistochemical Staining

Tissue samples for light microscopy were fixed in 4% formaldehyde and embedded in paraffin using routine procedures. Five-micrometer thick sections were cut from the tissue blocks and stained with hematoxylin-eosin.

For immunohistochemical staining the following primary antibodies were used: CD133/1 (AC133, 1 : 100, Miltenyi Biotec, Bergisch Gladbach, Germany) and CD44 (DF1485, 1 : 100, Dako, Glostrup, Denmark). No special pretreatment was used. The primary antibodies were visualized using the supersensitive streptavidin-biotin-peroxidase complex (Biogenex, San Ramon, CA). Appropriate positive and negative control slides were employed.

### 2.3. Semiquantitative Analysis of Slides

**Figure 1:**
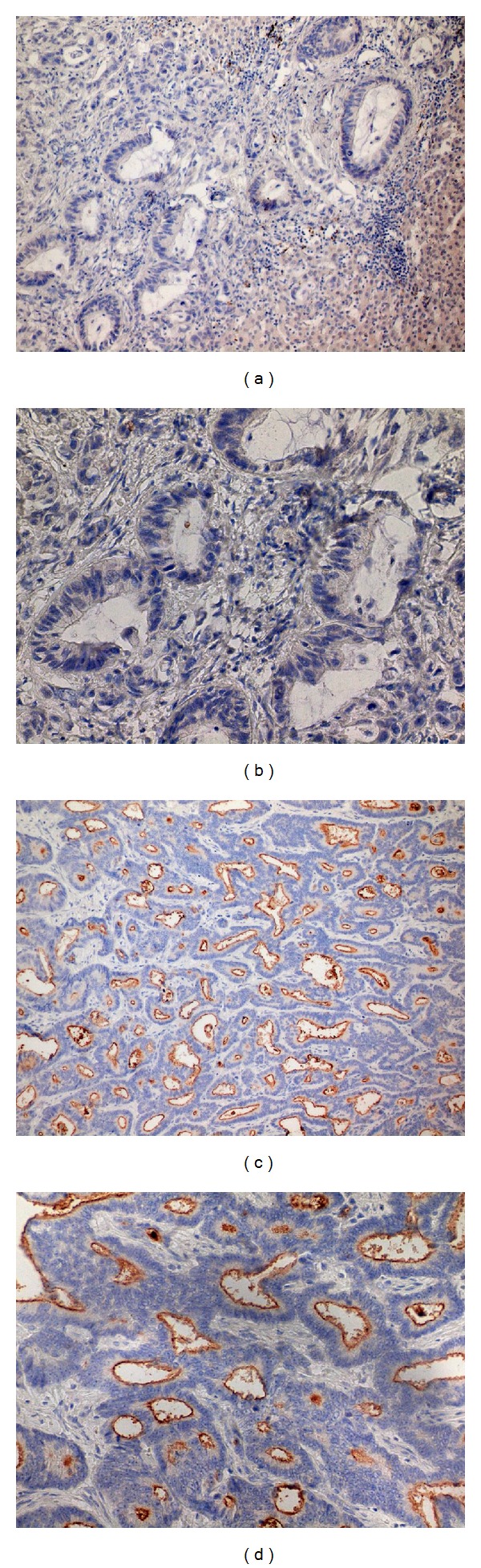
Expression of CD133. (a, b) Samples negative for CD133 in the lumen of tumor glands, (c, d) samples with positive CD133 staining on the apical portions of tumor cells. Magnification 200x (a, c) and 400x (b, d) of the same samples.

**Figure 2:**
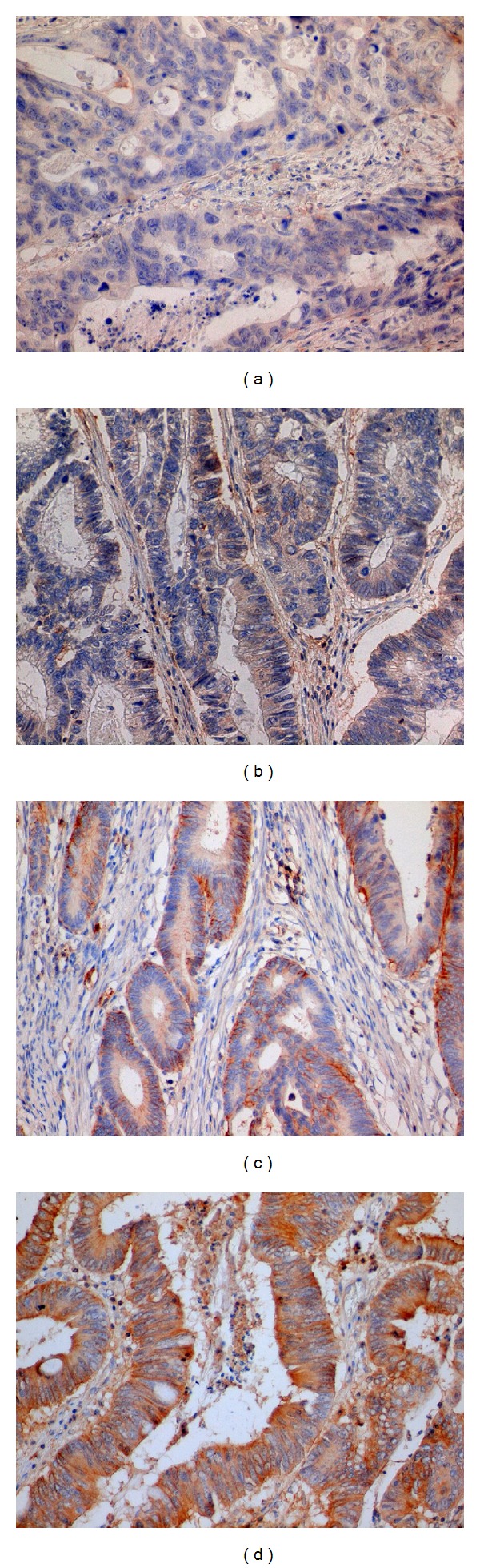
Example of samples with different levels of CD44 staining intensity. On our semiquantitative scale, samples were marked as intensity 0 (a), intensity 1 (b), intensity 2 (c), and intensity 3 (d). Magnification 400x.

A method based on previous study was used for analysis of slides [15]. All slides stained for CD44 and CD133 were analyzed independently by three trained researchers (Pavel Pitule, Miroslava Cedikova, and Jan Vojtisek). Tumors were localized using 10x objective and level of positivity on scale from 0 (negative) to 3 (highly positive) was assessed for CD44 staining. For CD133 we evaluated five microscopical fields using 40x objective and the percentage of CD133 positive tumor glands compared to all tumor glands in the view field were assessed. Positive staining of bile duct walls that should occur after every successful CD133 staining was used as an internal control of the staining process in case of liver metastasis samples. CLM slides with unstained bile ducts were excluded from the analysis. Examples of markers expression are summarized in Figures 1 and 2.

### 2.4. Statistical Analysis

CD44 and CD133 positivity assessments provided by the three researchers were averaged for every slide and in case of high variability of the scores the slide was reviewed by all of the three researchers. Resulting scores were on the scale from 0 to 3 for CD44 as described above and from 0 to 1 for CD133 expressing an average ratio of CD133 positive glands to all present glands.

Two overall survival (OS) times were defined for every patient, one from the time of the CRC surgery and the other from the time of the CLM surgery. Disease free interval (DFI) was calculated from the time of the CLM surgery to the time of metastases recurrence. The analyses of OS and DFI were performed using two-sample Kaplan-Meier method with Cox-Mantel test. The two samples (patient groups to be compared) were formed independently for each variable based on its median. Positive results (*P* < 0.05) were validated by Cox proportional hazards model with subsequent Chi-square test. Possible relations between tumor grading/staging and CD44/CD133 positivity were investigated using Mann-Whitney *U* test. Correlations between CD44/CD133 in CRC/CLM (all combinations) were explored using Spearman rank-order method. Statistical analysis was performed using the statistical software Statistica 10.0 (StatSoft, Inc. 2011, Tulsa, OK, USA).

## 3. Results

We have included 94 patients with primary and secondary CRC in our study. Samples with low quality staining were excluded from analyses. OS after CLM surgery at 1, 3, and 5 years was 88%, 65%, and 35%,respectively, and DFI at 1, 3, and 5 years was 38%, 16%, and 8%, respectively.

**Figure 3:**
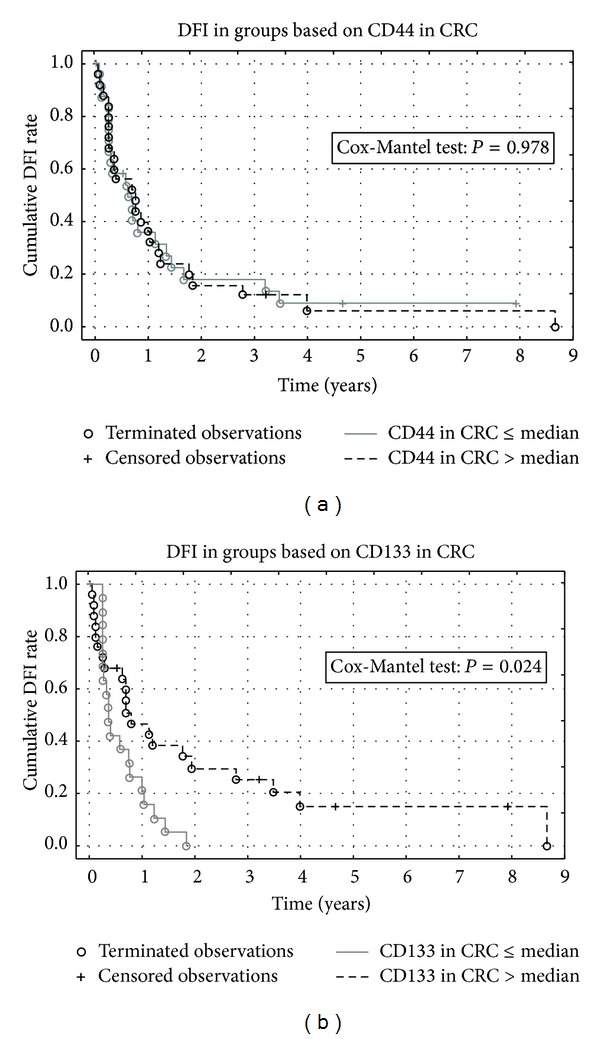
Kaplan-Meier curves comparing the levels of CD44 (a) or CD133 (b) staining intensity in primary colorectal cancer sample to the disease free interval.

**Figure 4:**
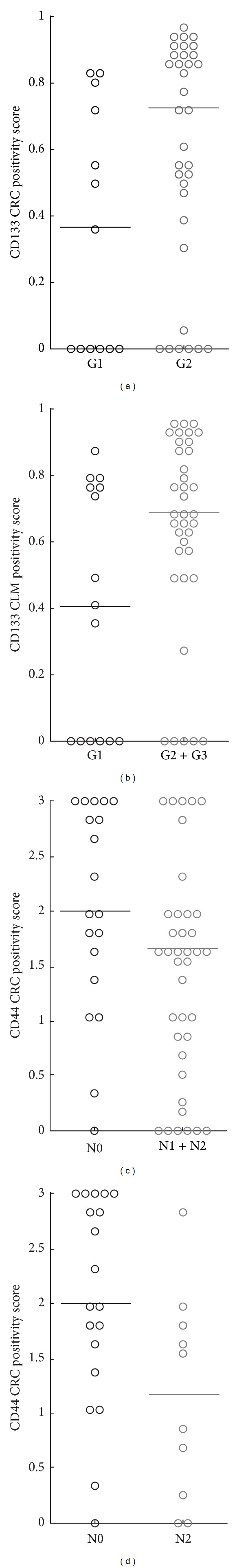
Comparison of the clinical data with the levels of markers abundance. (a) Difference of CD133 positivity in CRC based on tumor grade, (b) difference of CD133 positivity in CLM based on primary tumor grade, (c) comparison of CD44 intensity in CRC between N0 and N1 + N2 groups, and (d) comparison of CD44 intensity in CRC between N0 and N2 groups only.

We did not find any statistically significant effect of CD44 expression in CRC or CLM on either OS or DFI (Figure 3(a)). CD133 positivity over median in primary tumor was found to be a positive prognostic factor of DFI (Cox-Mantel *P* = 0.0244) (Figure 3(b)). This finding was confirmed by Cox proportional hazards model using the CD133 CRC score as a single independent variable (Chi-square *P* = 0.0137). CD133 positivity in CLM was not connected to any effect on OS or DFI (Cox-Mantel *P* = 0.3855). We identified differences in markers quantity based on grading, where CD133 in CRC was present in lower amount in G1 compared to G2 (Mann-Whitney *U* Test *P* = 0.0248) and CD133 in CLM had lower expression in G1 compared to combined G2 and G3 stage (Mann-Whitney *U* Test *P* = 0.0470) (Figures 4(a) and 4(b)). Comparison of studied markers with TNM classification revealed differences in CD44 in CRC depending on lymph node invasion—higher expression of CD44 was detected in N0 stage compared to combined N1 and N2 groups (Mann-Whitney *U* Test *P* = 0.0287) as well as N0 compared to N2 (Mann-Whitney *U* Test *P* = 0.0212) (Figures 4(c) and 4(d)).

Spearman correlation revealed a relationship between expression of CD133 in primary CRC and CLM (Spearman *R* = 0.5466, *P* = 0.00068).

## 4. Discussion

The concept of contribution of colorectal cancer stem cells to tumor development is widely accepted, but the relation of individual CSC markers expression to disease prognosis is still not completely clear [16]. In case of CD44, various splice variants differ in function and reports for CD44 in general usually fail to find any correlation with DFI or OS [17–19]. This was the case also for our set of patients, suggesting that use of CD44 as a single prognostic marker of CRC behavior is impossible. However, we observed a difference in CD44 expression when we stratified the patients according to tumor lymph node invasion with the data showing a decrease of CD44 expression in CRC in sequence from N0 to N2. Higher invasiveness of tumors with lower expression of CD44 into the lymph nodes can be related to weaker CD44 mediated binding to extracellular matrix [17].

CD133 was used as a first marker for identification of colorectal CSC [4, 5]. Immunohistochemical analysis of CD133 expression and its relevance to clinical and pathological features of CRC depends on sample type and size. Another problem is the posttranslation modification of CD133, which can mask AC133 epitope, which is the target for most antibodies against CD133 [20]. Some studies have shown that not the presence or absence of CD133 is important for CSCs identification, but that the abundance of CD133 protein can distinguish cells with different growth capacity [21]. Presented study did not assess the role of CD133 in cancer stem cell biology, but we wanted to find out whether it can be used as a marker providing new information to patients' prognosis. Large meta-analysis of CD133 expression in colorectal cancer confirmed that overexpression can be associated with several clinicopathological factors and can be used as an independent negative prognostic factor [22]. Surprisingly, the level of CD133 positivity had the opposite effect in our very confined group of patients as the statistical analysis revealed that higher levels of CD133 were associated to longer DFI. CD133 was described to be expressed in well and moderately differentiated tumors compared to undifferentiated tumor buds, which tend to be CD133 negative [23]. In metastatic CRC, CD133 expressing cells were described to be more often in G1/G0 phase of cell cycle than in S and G2/M phases [24]. Based on this information, CD133 cells can be considered to be those with low cycling rate and also those typical for tumors with better clinical outcome. These facts could be connected to positive prognostic effect of higher CD133 expression on DFI described in presented study.

Based on the described association between longer DFI and CD133 positivity in CRC but not in CLM, it is possible to speculate that the primary tumor has more important role in disease recurrence than liver metastasis.

## 5. Conclusion

Our study shows that in the field of cancer stem cells markers and their role on tumor behavior there is still a large space for further research. It seems that even commonly used CSCs marker CD133 can bear both negative and positive prognostic information depending on the clinical specification of studied patients and therefore there is a need for new studies aiming at describing the effect and role of CSCs markers in well-defined sets of samples. Also testing of combination of several markers can be of particular importance. It would be of great importance to fully understand the biology of individual proteins used as markers, because it can provide a new point of view on the seemingly contradictory results from individual studies. Generally, if applied with underlying understanding mentioned above, cancer stem cell markers can bring valuable information to patients' prognosis and can help to modify diagnostic and treatment strategy.

## Figures and Tables

**Table 1:** Description of set of patients used for the study.

Total number	94
Gender (males/females)	57/37
Age at primary surgery (years)	
Median	61.9
Interquartile range	12.4
Age at liver surgery (years)	
Median	63.2
Interquartile range	11.9
Tumor size (T)	
T1	3
T2	3
T3	73
T4	9
Unknown	6
Lymph nodes involvement (N)	
N0	27
N1	37
N2	16
Unknown	14
M classification	
M0	45
M1	49
Grade	
G1	19
G2	53
G3	13
Unknown	9

## References

[1] Bonnet D, Dick JE (1997). Human acute myeloid leukemia is organized as a hierarchy that originates from a primitive hematopoietic cell. *Nature Medicine*.

[2] Al-Hajj M, Wicha MS, Benito-Hernandez A, Morrison SJ, Clarke MF (2003). Prospective identification of tumorigenic breast cancer cells. *Proceedings of the National Academy of Sciences of the United States of America*.

[3] Collins AT, Berry PA, Hyde C, Stower MJ, Maitland NJ (2005). Prospective identification of tumorigenic prostate cancer stem cells. *Cancer Research*.

[4] Ricci-Vitiani L, Lombardi DG, Pilozzi E (2007). Identification and expansion of human colon-cancer-initiating cells. *Nature*.

[5] O’Brien CA, Pollett A, Gallinger S, Dick JE (2007). A human colon cancer cell capable of initiating tumour growth in immunodeficient mice. *Nature*.

[6] Li C, Heidt DG, Dalerba P (2007). Identification of pancreatic cancer stem cells. *Cancer Research*.

[7] Gottschling S, Schnabel PA, Herth FJF, Herpel E (2012). Are we missing the target? Cancer stem cells and drug resistance in non-small cell lung cancer. *Cancer Genomics Proteomics*.

[8] Aguilar-Gallardo C, Simón C (2013). Cells, stem cells, and cancer stem cells. *Seminars in Reproductive Medicine*.

[9] Ishii H, Iwatsuki M, Ieta K (2008). Cancer stem cells and chemoradiation resistance. *Cancer Science*.

[10] Friedman MD, Jeevan DS, Tobias M, Murali R, Jhanwar-Uniyal M (2013). Targeting cancer stem cells in glioblastoma multiforme using mTOR inhibitors and the differentiating agent all-trans retinoic acid. *Oncology Reports*.

[11] Dalerba P, Dylla SJ, Park I-K (2007). Phenotypic characterization of human colorectal cancer stem cells. *Proceedings of the National Academy of Sciences of the United States of America*.

[12] Vermeulen L, Todaro M, De Sousa Mello F (2008). Single-cell cloning of colon cancer stem cells reveals a multi-lineage differentiation capacity. *Proceedings of the National Academy of Sciences of the United States of America*.

[13] Huang EH, Hynes MJ, Zhang T (2009). Aldehyde dehydrogenase 1 is a marker for normal and malignant human colonic stem cells (SC) and tracks SC overpopulation during colon tumorigenesis. *Cancer Research*.

[14] Shmelkov SV, Butler JM, Hooper AT (2008). CD133 expression is not restricted to stem cells, and both CD133^+^ and CD133^−^ metastatic colon cancer cells initiate tumors. *Journal of Clinical Investigation*.

[15] Liska V, Vycital O, Daum O (2012). Infiltration of colorectal carcinoma by S100^+^ dendritic cells and CD57^+^ lymphocytes as independent prognostic factors after radical surgical treatment. *Anticancer Research*.

[16] Ren F, Sheng WQ, Du X (2013). CD133: a cancer stem cells marker, is used in colorectal cancers. *World Journal of Gastroenterology*.

[17] Galizia G, Gemei M, Del Vecchio L (2012). Combined CD133/CD44 expression as a prognostic indicator of disease-free survival in patients with colorectal cancer. *Archives of Surgery*.

[18] Li XD, Ji M, Wu J, Jiang JT, Wu CP (2013). Clinical significance of CD44 variants expression in colorectal cancer. *Tumori*.

[19] Langan RC, Mullinax JE, Ray S (2012). A pilot study assessing the potential role of non-CD133 colorectal cancer stem cells as biomarkers. *Journal of Cancer*.

[20] Kemper K, Sprick MR, De Bree M (2010). The AC133 epitope, but not the CD133 protein, is lost upon cancer stem cell differentiation. *Cancer Research*.

[21] Liao Y, Hu X, Huang X, He C (2010). Quantitative analyses of CD133 expression facilitate researches on tumor stem cells. *Biological and Pharmaceutical Bulletin*.

[22] Chen S, Song X, Chen Z (2013). CD133 expression and the prognosis of colorectal cancer: a systematic review and meta-analysis. *PLoS ONE*.

[23] Horst D, Kriegl L, Engel J, Kirchner T, Jung A (2008). CD133 expression is an independent prognostic marker for low survival in colorectal cancer. *British Journal of Cancer*.

[24] Gharagozloo M, Mirzaei HR, Bagherpour B (2012). Cell cycle analysis of the CD133^+^ and CD133^−^ cells isolated from human colorectal cancer. *Journal of Cancer Research and Therapeutics*.

